# Preparation of CNT/CNF/PDMS/TPU Nanofiber-Based Conductive Films Based on Centrifugal Spinning Method for Strain Sensors

**DOI:** 10.3390/s24124026

**Published:** 2024-06-20

**Authors:** Shunqi Mei, Bin Xu, Jitao Wan, Jia Chen

**Affiliations:** 1 Hubei Digital Textile Equipment Key Laboratory, Wuhan Textile University, Wuhan 430073, China; sqmei@wtu.edu.cn (S.M.); 13061159209@163.com (B.X.); 13636062780@163.com (J.C.); 2 The Advanced Textile Technology Innovation Center (Jianhu Laboratory), Shaoxing 312000, China; 3 School of Mechanical & Electrical Engineering, Xi’an Polytechnic University, Xi’an 710048, China

**Keywords:** flexible sensors, centrifugal spinning method, fiber-based conductive films, PDMS modification, blended fillers

## Abstract

Flexible conductive films are a key component of strain sensors, and their performance directly affects the overall quality of the sensor. However, existing flexible conductive films struggle to maintain high conductivity while simultaneously ensuring excellent flexibility, hydrophobicity, and corrosion resistance, thereby limiting their use in harsh environments. In this paper, a novel method is proposed to fabricate flexible conductive films via centrifugal spinning to generate thermoplastic polyurethane (TPU) nanofiber substrates by employing carbon nanotubes (CNTs) and carbon nanofibers (CNFs) as conductive fillers. These fillers are anchored to the nanofibers through ultrasonic dispersion and impregnation techniques and subsequently modified with polydimethylsiloxane (PDMS). This study focuses on the effect of different ratios of CNTs to CNFs on the film properties. Research demonstrated that at a 1:1 ratio of CNTs to CNFs, with TPU at a 20% concentration and PDMS solution at 2 wt%, the conductive films crafted from these blended fillers exhibited outstanding performance, characterized by electrical conductivity (31.4 S/m), elongation at break (217.5%), and tensile cycling stability (800 cycles at 20% strain). Furthermore, the nanofiber-based conductive films were tested by attaching them to various human body parts. The tests demonstrated that these films effectively respond to motion changes at the wrist, elbow joints, and chest cavity, underscoring their potential as core components in strain sensors.

## 1. Introduction

Strain sensors are extensively employed in smart clothing, electronic skin, and wearable medical devices, leveraging their ability to convert physical motions such as compression, bending, stretching, shearing, stress, and strain into electrical signals [1,2,3,4]. Flexible conductive films are crucial for transmitting these signals, with their performance directly influencing the quality of strain sensors. Developing such materials that are lightweight, highly flexible, and capable of adapting to diverse environmental changes while maintaining stable conductivity remains the principal challenge in this field [5,6,7].

Numerous studies have focused on the preparation of flexible conductive films. For example, Qiu et al. [8] developed stretchable transparent conductive films (STCFs) utilizing silver nanowires (AgNWs) via a hot pressing technique; these films are applicable in flexible circuits and tension sensors. Zhou et al. [9] fabricated flexible MXene/AgNW-PVA transparent conductive films. Wang et al. [10] utilized a hybrid method to develop graphene–thermopolyurethane (G-TPU) flexible conductive films characterized by electrical conductivity, thermal conductivity, and self-healing properties. Zhai et al. [11] employed the vacuum filtration method to fabricate AgNW-mixed cellulose ester flexible transparent conductive films exhibiting good mechanical stability. Lynch et al. [12] proposed the use of a graphene–natural rubber hybrid ink to print strain films composed of graphene composites, which exhibit high electrical conductivity. Cao et al. [13] prepared conductive graphene thin films via electrochemical vapor deposition, which boast high sensitivity and a wide pressure range. Sun et al. [14] fabricated a PANI-CNTs hydrogel using a 3D printing technology sensor, which possesses excellent mechanical properties suitable for the continuous monitoring of stress, strain, temperature, and human motion.

In comparison to the flexible conductive films fabricated using the aforementioned methods, fiber-based conductive films exhibit significant advantages in the field of flexible electronics and wearable devices attributable to their flexibility, stretchability, lightweight, breathability, high sensitivity from a high surface area-to-volume ratio, strength, durability, cost-effectiveness, ease of processing, and multifunctionality [15,16,17,18]. Currently, the mainstream methods for fabricating fiber-based conductive films encompass dry–wet spinning, electrospinning, and centrifugal spinning [19,20,21].

Wet and dry spinning are common spinning techniques. In these processes, the polymer is dissolved in a solvent to create a spinning solution. This solution is then extruded through a spinneret and carried out in air, a method known as dry spinning. Subsequently, the initially formed fibers are immersed in a coagulation bath, where the polymer solidifies, and the final fibers are formed, a procedure referred to as wet spinning [22]. Numerous studies focus on the use of wet and dry spinning in the preparation of conductive films; for example, Laia et al. [23] employed dry–wet spinning techniques to develop PET/graphene nanocomposite fibers for fabricating electrically conductive fabrics, exhibiting good mechanical stability and electrical conductivity. Gao et al. [24] fabricated a white electrically conductive fiber utilizing P3HB4HB polymers and ATO@TiO2 nanoparticles through the dry–wet spinning technique. This fiber notably exhibits over 1000% stretchability alongside good electrical conductivity, making it suitable for smart textiles.

Dry–wet spinning consumption of a large amount of solvent not only escalates costs but also poses potential environmental pollution. In comparison with electrospinning and centrifugal spinning, dry–wet spinning is limited to producing micron-sized fibers and is incapable of generating nanosized fibers. Such a limitation diminishes its applicability in contexts demanding finer fibers [25]. Research indicates that a reduction in fiber diameter to the nanometer scale substantially enhances film performance [26]. Consequently, methods such as electrospinning and centrifugal spinning, which are capable of producing nanoscale fibers, have garnered significant attention.

The electrospinning process involves subjecting a polymer solution to high voltage; upon reaching a critical level, the solution ejects a jet stream through a nozzle, which is then deposited on a collector plate to form nanofibers [27]. Electrospinning boasts a wide array of applications in the research of conductive films. For example, Li et al. [28] reported a highly permeable and stretchable conductor based on electrostatically spun fluoroelastomer fiber mats. Fiber properties were enhanced through pre-stretching in an electric field. Yoon et al. [29] explored the impact of nanofiber film surface coverage on conductivity, utilizing indium tin oxide (ITO) nanofibers prepared via electrospinning. Liu et al. [30] utilized electrospinning and spraying techniques to fabricate an Epoxy/NBR electrospun fiber with AgNW/PU composite coating, resulting in highly elastic conductive fiber electrodes. These electrodes maintained low resistance at 40% strain and withstood 200 expansion/release cycles at 30% strain, showcasing exceptional stability. Liu et al. [31] employed Y-connectors with barbs as spinnerets to electrospin camphorated acid (CSA) doped polyaniline (PANI) and polyethylene oxide (PEO) into side-by-side bipartite electrodes using electrostatic spinning. The resulting side-by-side bicomponent fibers exhibited excellent ductility and low relative resistivity.

Fiber-based conductive films, prepared via the electrospinning method, have undergone extensive study. However, their slow production, complex processes, high voltage requirements, and significant equipment cost and maintenance constrain large-scale production [32]. The centrifugal spinning method offers significant potential for the preparation of nanofiber films, attributed to its high yield, straightforward design, absence of high-pressure requirements, and environmental sustainability compared to other spinning techniques [33,34]. The centrifugal spinning method [35] involves injecting a polymer solution into the tank of the centrifugal spinning equipment and utilizing a high-speed rotating force to eject the solution from the nozzle, thus forming nanofibers. These fibers are subsequently collected and compressed to form the nanofiber film. For instance, Luo et al. [36] utilized centrifugal spinning to fabricate CNT/PU nanofiber conductive films characterized by robust electrical conductivity and elasticity. These films demonstrated exceptional electrical conductivity when stretched and maintained strong durability and stability through over 600 cycles. This outcome further validates the centrifugal spinning method’s potential for nanofiber conductive film fabrication, with its high productivity offering the prospect of mass-producing flexible conductive films.

The selection of both the fiber substrate and the conductive filler, in addition to the preparation method, is crucial in determining the performance of conductive films. Polyurethane, a segmented polymer, is composed of a soft segment and a hard segment. The soft segment, comprising long-chain polyols, imparts flexibility and elasticity to the material, while the hard segment, resulting from a condensation reaction between isocyanate and short-chain polyols, provides mechanical strength and chemical stability [37]. Such a structure renders polyurethane an ideal substrate for fibers. Jiang et al. [38] fabricated fibrous TPU films via electrospinning, subsequently saturating them with ionic liquids (IL). The resultant stretchable sensors exhibited rapid response times, ultra-low detection limits, and an expansive sensing range. Wang et al. [39] developed TPU/CB fiber membranes through electrospinning, noted for their high sensitivity. The influence of the electrospinning collection device’s rotational speed on both the network structure of the stereoscopic scaffold and the fiber diameter of TPU/CB composites was examined.

Carbon nanotubes have good remarkable electron transport properties and mechanical strength and flexibility due to their unique one-dimensional structure, and they are common conductive fillers for a wide range of applications in the field of flexible electronics [40]. Yu et al. [41] reported on a flexible strain sensor comprising a TPU fiber film decorated with CNTs, prepared using electrostatic spinning technology. Luo et al. [42] fabricated a PU fiber mat via electrospinning and subsequently decorated it with carbon nanotubes (CNTs) using ultrasonic cavitation treatment, resulting in a PU/CNT film suitable for use as a wearable thin-film adapter. This process yielded PU/CNT films, which are applicable as wearable thin-film heaters, strain sensors, and stretchable supercapacitor electrodes for energy storage. Carbon nanofibers show great potential for application in high-performance flexible electronics due to their high electrical conductivity, excellent mechanical strength, lightweight flexibility, high thermo-chemical stability, and ease of processing. Huang et al. [43] fabricated a titanium carbide (TiC)/carbon nanofiber (CNF) composite electric heating film through electrospinning, which exhibited high thermal conversion efficiency and outstanding electro-thermal cycle stability. Ren et al. [44] employed electrospinning to deposit polyacrylonitrile nanofibers onto graphene films, resulting in a-PAN/G films characterized by high transparency and superior electrical and mechanical properties. Considering the unique advantages of both CNTs and CNFs, blending these nanomaterials could not only create a more complex conductive network at the microscopic scale but also significantly enhance the mechanical properties of the films. Such a microstructure offers optimized conductive pathways at the nanoscale, crucial for boosting the films’ sensitivity and sensing performance. Thus, the combined use of CNT and CNF fillers represents a promising research avenue.

To enhance the service life, corrosion resistance, and hydrophobicity of conductive films, PDMS, known for its low surface energy, robust chemical stability, and superior mechanical properties and flexibility, has gained prominence. Duan et al. [45] fabricated a 3D conductive network composed of polyurethane fibers (PUFs) and poly(3,4-ethylenedioxythiophene) (PEDOT) coatings using electrospinning and in situ interfacial polymerization. Subsequently, they backfilled this network with PDMS to create a 3D conductor notable for its excellent mechanical durability and stretchability. Wang et al. [46] coated CNTs onto the surface of electrostatically spun TPU nanofibers via ultrasonication, subsequently modifying them with PDMS to develop TPU/CNTs/PDMS superhydrophobic integrated strain sensors. However, the costs and efficiency of electrostatic spinning have hindered mass production.

Based on the above analysis, this paper proposes an innovative method: employing centrifugal spinning to prepare conductive thin films based on polydimethylsiloxane (PDMS)-modified carbon nanotubes (CNTs), carbon nanofibers (CNFs), and thermoplastic polyurethane (TPU) nanofibers. TPU exhibits exceptional elasticity and flexibility, maintaining stable performance across a broad range of strains. CNTs, notable for their excellent electron transport properties and mechanical strength, significantly enhance the sensing sensitivity and serve as conductive fillers in composites. CNFs offer remarkable thermal stability and mechanical properties, providing additional structural support to the composite films and further improving their overall performance. PDMS, utilized as a modifier, demonstrates outstanding flexibility and ease of handling, facilitating the enhancement of the composite conductive films’ performance. In this study, CNTs and CNFs were introduced as blended conductive fillers, aimed at further enhancing the performance of the conductive films. The centrifugal spinning method was employed to prepare nanofiber-based conductive films, laying the foundation for subsequent large-scale production. We systematically investigated the electrical conductivity, elongation at break, hydrophobicity, corrosion resistance, and tensile cycling stability of these conductive films, particularly focusing on how the ratio of CNTs to CNFs affects their overall performance. Furthermore, this study explores the potential applications of these nanofiber-based conductive films in developing novel sensors, particularly for monitoring human motion, including joint movement and respiratory rate monitoring.

## 2. Materials and Methods

### 2.1. Materials

The primary experimental materials comprised thermoplastic polyurethane (TPU) particles (Elastollan 1185A) obtained from BASF Polyurethanes Specialties (China) Co., Ltd., Shanghai, China; carboxylated multi-walled carbon nanotube water dispersions (CNTs) and carbon nanofiber (CNF) powders sourced from Jiangsu Xianfeng Nanomaterials Technology Co., Ltd., Wuxi, China; polydimethylsiloxane (PDMS Sylgard 184) acquired from Dow Corning Corporation in the USA, Midland, MI, USA; and chemically pure dimethylformamide (DMF) and n-heptane procured from Sinopharm Chemical Reagent Co., Ltd., Shanghai, China.

### 2.2. Preparation of TPU Nanofiber Films

**Figure 1:**
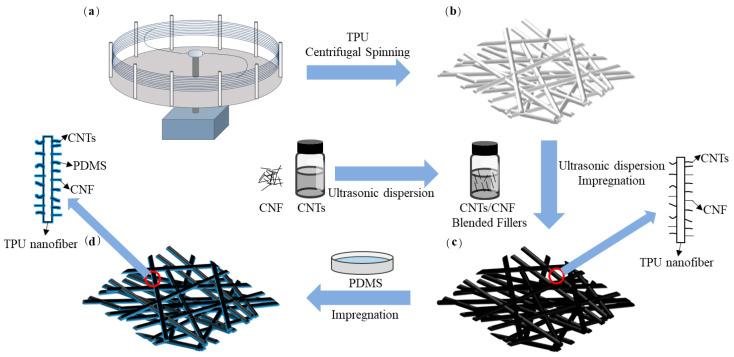
Flow chart for the preparation of CNT/CNF/PDMS/TPU nanofiber-based conductive films: (**a**) schematic diagram of the centrifugal spinning equipment; (**b**) TPU nanofiber film; (**c**) CNT/CNF/TPU nanofiber film; (**d**) CNT/CNF/PDMS/TPU nanofiber-based conductive film.

Initially, the TPU solution was prepared by dissolving thermoplastic polyurethane pellets in DMF and stirred at 80 °C for 8 h using a magnetic stirrer. Preliminary tests demonstrated that a 20% TPU solution concentration resulted in large, uniform fibers via centrifugal spinning, facilitating easy collection. The TPU solution was then injected into centrifugal spinning equipment, designed and manufactured by our research team, as illustrated in Figure 1a. Rotational speed was maintained at 4000 rpm, with the distance between the spinneret and the collection net set to 30 cm. Fibers were collected on aluminum foil at 7 min intervals, achieving the desired thickness of TPU nanofiber film after five collections, depicted in Figure 1b. Subsequently, TPU nanofiber films with a thickness of 0.1 mm were produced by compressing under a 2 kg iron plate and subsequently drying in an oven at 65 °C for 2 h to eliminate residual solvents. Finally, the film was cut into samples measuring 4 cm in length and 1 cm in width.

### 2.3. Preparation of CNT/CNF/TPU Nanofiber-Based Conductive Films

Carbon nanotubes (CNTs) are coaxially coiled by multiple seamless tubular structures, and carbon nanofibers (CNFs) exhibit a fibrous, long, and thin arrangement of carbon atoms, wherein carboxylated multi-walled carbon nanotube (CNT) aqueous dispersions are better dispersed when blended with carbon nanofiber powders due to the unique carboxyl (-COOH) functional group. The aqueous dispersion of carboxylated multi-walled carbon nanotubes (CNTs) was first added proportionally to deionized water, and then carbon nanofiber (CNF) powders were added proportionally to deionized water and sonicated for 30 min using an ultrasonic disperser operating at a frequency of 20 kHz and a power of 240 W. Five sets of uniformly dispersed CNT/CNF-blended conductive filler solutions were prepared in the ratios of CNT = 4:1, 3:2, 1:1, 2:3, and 1:4. Subsequently, cut thermoplastic polyurethane (TPU) films were immersed in these solutions for 10 h. Subsequently, the films were removed from the dispersion solution, rinsed with deionized water, and dried. The films were then re-immersed in the co-conductive filler solution and ultrasonically treated for one hour to enhance the adhesion of CNTs and CNFs on the TPU films. Finally, the films were removed, cleaned, and dried to produce the final CNT/CNF/TPU films, as illustrated in Figure 1c.

### 2.4. Preparation of CNT/CNF/PDMS/TPU Nanofiber-Based Conductive Films

The PDMS prepolymer was prepared by mixing basic components and curing agents at a mass ratio of 10:1. The PDMS solution was formulated by blending the PDMS prepolymer with n-heptane, followed by stirring for 30 min with a magnetic stirrer. Preliminary testing indicated an improved leaching effect at a PDMS concentration of 2 wt% [46]. Consequently, for the studies detailed in this paper, CNT/CNF/TPU nanofiber-based films were submerged in a 2 wt% PDMS solution for 2 h. The films were then removed and dried in a vacuum oven at 65 °C for 2 h to facilitate the evaporation of the residual solvent and curing of PDMS. Ultimately, the CNT/CNF/PDMS/TPU nanofiber-based conductive films were produced, as illustrated in Figure 1d.

### 2.5. Testing and Characterization

Microstructural observations were made using a JSM-7800 scanning electron microscope (JEOL, Tokyo, Japan). Fourier-transform infrared spectroscopy (FTIR) measurements were conducted using an FTIR-7600 (Lambda Scientific, Edwardstown, NSW, Australia) spectrometer in an attenuated total reflection (ATR) mode, covering a measurement range from 400 to 4000 cm^−1^. X-ray diffraction (XRD) analysis was performed using a Panaco Empyrean Sharp X-ray diffractometer (Panaco, Alemlo, The Netherlands). Thermal stability was assessed through thermogravimetric analysis using an STA300 Thermogravimetric Analyzer (Hitachi, Tokyo, Japan). Conductivity was determined by averaging measurements from at least five distinct positions on each sample, utilizing a four-probe resistance tester (ST2253y, Suzhou Jingge Electronics Co., Ltd., Suzhou, China). Elongation at break for the conductive film samples was measured with a dynamic fabric resistance tester (FZ01, Shanghai Afes Precision Instruments Co., Ltd., Shanghai, China).

Contact angle measurements were performed using an OCA20 contact angle meter (Dataphysics, Stuttgart, Germany) by applying 5 μL of deionized water to the film’s surface and recording the contact angle after 30 s. Each sample’s contact angle (CA) value was averaged from three distinct surface positions, providing an assessment of the film’s hydrophobicity.

Cyclic tensile testing was conducted on the conductive films using a dynamic fabric resistance tester (FZ01 Shanghai Afes Precision Instruments Co., Ltd.) and a digital oscilloscope (Tektronix, Biverton, OR, USA), facilitating analysis of electrical resistance changes, tensile rate changes, and the calculation of the sensitive strain coefficient.

Additionally, the cyclic tensile test was also performed for 800 cycles to check the cyclic stability of the conductive films. Additionally, human motion was monitored using flexible sensors fabricated from CNT/CNF/PDMS/TPU nanofiber-based films. A digital oscilloscope recorded changes in the films’ resistance, confirming their viability for wearable device applications.

## 3. Results

### 3.1. Morphological Characterization

**Figure 2:**
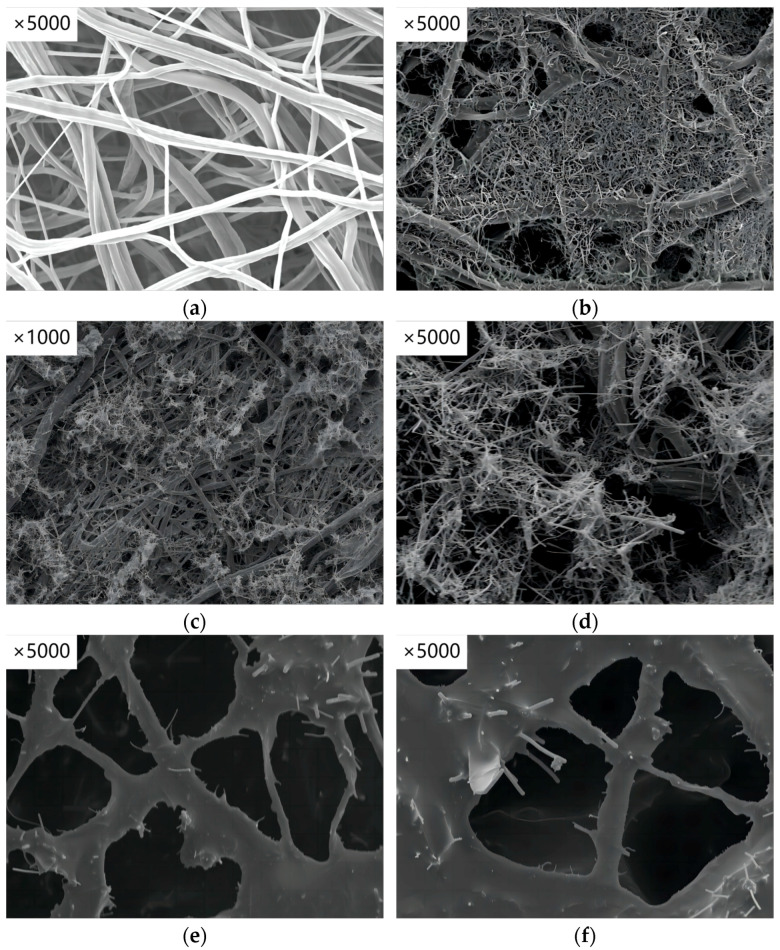
SEM images of nanofiber-based conductive films: (**a**) Pure TPU film; (**b**) CNT/TPU film; (**c**,**d**) CNT/CNF/TPU films; (**e**,**f**) CNT/CNF/PDMS/TPU films.

The SEM image in Figure 2a reveals that the TPU nanofibers are interwoven, resulting in a robust three-dimensional reticulated pore structure. This structure offers an extensive surface area for the anchoring of conductive fillers. Figure 2b depicts a CNT/TPU nanofiber-based conductive film, highlighting that the CNTs are effectively anchored onto the TPU nanofibers following ultrasonic dispersion impregnation. Figure 2c,d illustrate the morphology of CNT/CNF/TPU films at magnifications of 1000× and 5000×, respectively. The images demonstrate that the ultrasonic dispersion treatment enables CNTs to anchor onto CNFs, creating a solid connection. Here, CNFs act akin to a backbone, establishing a robust foundation for the conductive pathway. The morphological characterization of both CNT/CNF/TPU and CNT/CNF/PDMS/TPU films is presented. Figure 2e,f show that PDMS incorporation effectively bonds CNTs and CNFs, thereby enhancing the interfacial adhesion between the conductive components and the fiber substrate.

### 3.2. Conductive Film Composition and Thermal Stability

**Figure 3:**
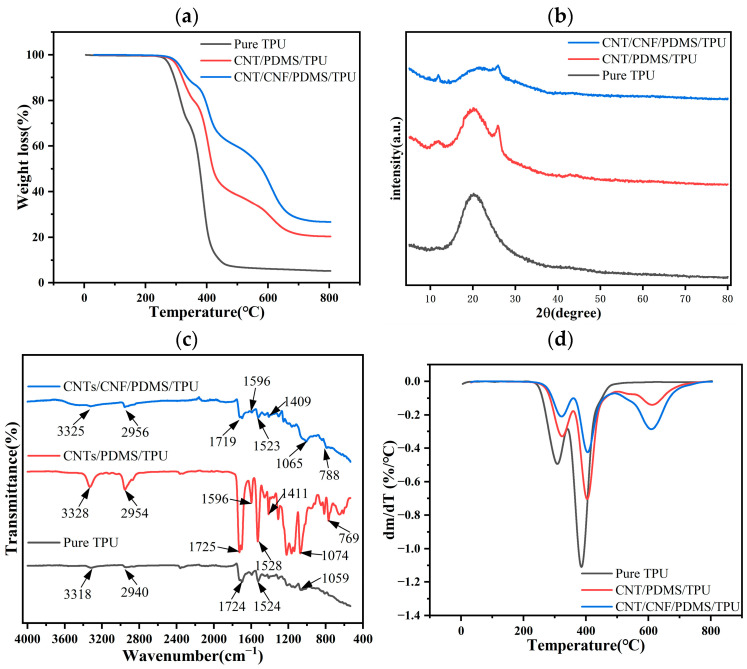
(**a**) FTIR spectra; (**b**) XRD diffractograms; (**c**) TGA curves; and (**d**) DTG curves for pure TPU film, CNT/PDMS/TPU film, and CNT/CNF/PDMS/TPU film.

To confirm the adsorption of CNTs, CNFs, and PDMS onto the TPU nanofiber substrate films, Fourier-transform infrared (FTIR) spectroscopy was conducted on pure TPU films, CNT/PDMS/TPU films, and CNT/CNF/PDMS/TPU films. As shown in Figure 3a, the IR spectra of the pure TPU nanofiber films showed absorption peaks due to N-H bond stretching vibration at 3318 cm^−1^ and 1524 cm^−1^, C-H stretching vibration at 2940 cm^−1^, carbonyl group at 1724 cm^−1^, and C-O-C ether bond stretching. Following the ultrasonic dispersion anchoring of CNTs onto TPU nanofiber films, the positions of the absorption peaks in the infrared spectra of the CNT/PDMS/TPU films changed to 3328 cm^−1^, 2954 cm^−1^, 1725 cm^−1^, 1528 cm^−1^, and 1074 cm^−1^, respectively, and the C=C double-bond stretching vibration was observed at 1596 cm^−1^. PDMS addition introduced new characteristic peaks; a 1411 cm^−1^ peak from silicon-methyl group (-CH3) vibrations, and a 769 cm^−1^ peak from Si-O-Si bond vibrations. Upon blending CNFs with CNTs and anchoring them onto TPU films, no new peaks emerged due to the similar carbon composition of CNFs and CNTs. However, original peak shifts were observed at 3325 cm^−1^, 2956 cm^−1^, 1719 cm^−1^, 1523 cm^−1^, 1065 cm^−1^, and 788 cm^−1^. These spectral changes indicate the successful adsorption of CNTs, CNFs, and PDMS onto the TPU nanofiber substrate film.

According to the X-ray diffraction (XRD) patterns shown in Figure 3b, the XRD profiles of pure thermoplastic polyurethane (TPU) film, carbon nanotube/polydimethylsiloxane/TPU (CNTs/PDMS/TPU) film, and carbon nanotube/carbon nanofiber/polydimethylsiloxane/TPU (CNT/CNF/PDMS/TPU) film all exhibit characteristic diffraction peaks of TPU at 20°. However, the shapes and intensities of these peaks in composites containing CNTs and CNFs differ significantly. Both the CNT/PDMS/TPU and CNT/CNF/PDMS/TPU films display new diffraction peaks at 13° and 26°, respectively. XRD is primarily employed to analyze the structure of crystalline materials, as the orderly arrangement of crystals generates specific diffraction patterns. Since PDMS is an amorphous polymer, its molecular structure results in a broad diffraction peak rather than a sharp peak in XRD spectra, typically observed in the low-angle region of 2θ [47]. Consequently, the peak at 13° is attributed to PDMS, while the peak at 26° is ascribed to both CNTs and CNFs, with the latter displaying characteristics similar to CNTs in XRD spectra [48]. Differences in the peak shape, intensity, and position between these films are primarily due to the interactions between CNTs and CNFs and their differential compatibility and dispersion within the TPU matrix.

Thermogravimetric analysis (TGA) was employed to analyze the thermal stability of the CNT/CNF/PDMS/TPU fiber-based conductive films. Figure 3a,b display the TGA and corresponding DTG curves for both the pure TPU fiber-based films and their composites. Following thermal decomposition, the residue weight percentages were calculated as 5.3%, 20.3%, and 26.5% for the pure TPU film, CNT/PDMS/TPU film, and CNT/CNF/PDMS/TPU film, respectively. The initial decomposition temperatures for the pure TPU films, CNT/PDMS/TPU films, and CNT/CNF/PDMS/TPU films were consistently near 290 °C, as indicated by the TGA results. Moreover, the TGA curves for the CNT/PDMS/TPU film and CNT/CNF/PDMS/TPU film significantly shifted toward the higher temperature region. The DTG curves reveal that the maximum decomposition temperatures for the pure CNT/PDMS/TPU film and CNT/CNF/PDMS/TPU film are 387.8 °C, 404.4 °C, and 406.9 °C, respectively. This demonstrates that the maximum decomposition temperature of the conductive films prepared in this study exceeds the 325 °C reported for CNT/PU films in another study [36], underscoring the effective improvement in thermal stability due to the addition of PDMS and CNF.

### 3.3. Film Electrical Conductivity

**Figure 4:**
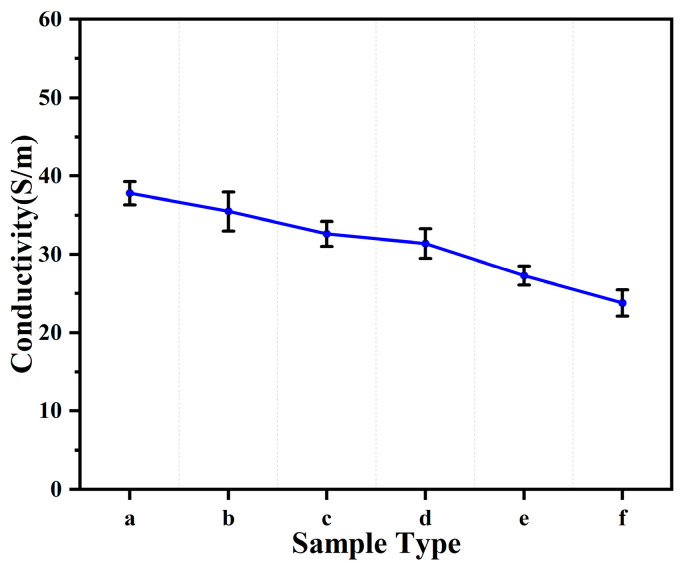
Conductivity of different thin film samples. (a) CNTs/PDMS/TPU; (b) CNTs:CNFs = 4:1/PDMS/TPU; (c) CNTs:CNFs = 3:2/PDMS/TPU; (d) CNTs:CNFs = 1:1/PDMS/TPU; (e) CNTs:CNFs = 2:3/PDMS/TPU; (f) CNTs:CNFs = 1:4/PDMS/TPU.

In order to investigate the effect of the blended filler ratio of CNTs and CNFs on the electrical conductivity of fiber-based conductive films, the conductivity was measured at five different points on the conductive films by using a four-probe resistance tester, and the average conductivity of five groups of samples was obtained, as shown in Figure 4. It can be seen that the conductivity decreased after the addition of CNFs, which is due to the fact that CNTs have a nearly perfect hexagonal carbon atom arrangement and fewer defects, giving them very high structural symmetry and integrity on the nanoscale. The one-dimensional structure of CNTs provides an excellent electron transport channel, allowing the electrons to move along the direction of the tube axis inside the carbon nanotubes without any obstacles, which makes the conductivity of CNTs superior to that of CNFs. When the ratios of the blended conductive fillers are 4:1, 3:2, and 1:1, the conductivities of the films measure 35.5 S/m, 32.6 S/m, and 31.4 S/m, respectively. Despite a slight decrease, this change is not significant, suggesting that the mixture of CNTs and CNFs does not markedly affect the conductivity within these ratios. Although conductivity decreased overall with increasing proportions of CNFs, the decrease was not sharp and conductivity remained high, indicating that CNTs and CNFs, as blended conductive fillers, can form an effective conductive path. Compared to the conductivity of TPU/CNT/PDMS elastic nanofiber composites reported elsewhere (27.1 S/m) [46], the films in this study, with blended filler ratios of 4:1, 3:2, and 1:1, exhibited superior conductivity. This confirms the effectiveness of the method and underscores the potential to tune the ratio of CNTs to CNFs to optimize electrical conductivity properties.

### 3.4. Film Elongation at Break

**Figure 5:**
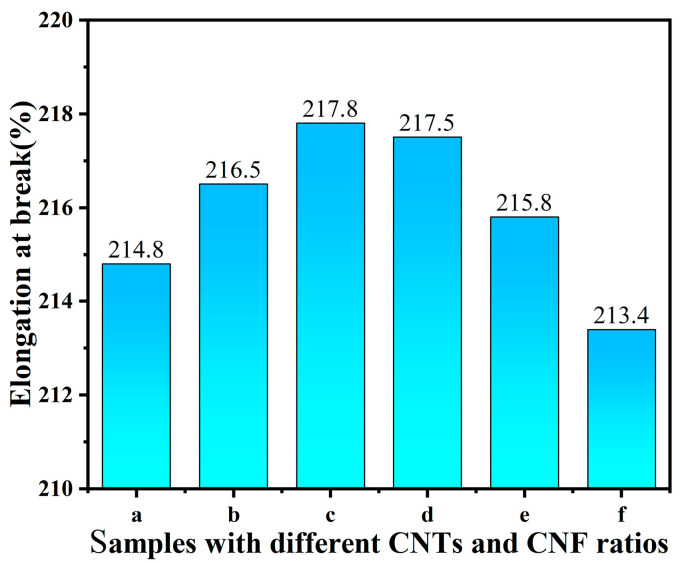
Elongation at break of different film samples. (a) CNTs/PDMS/TPU; (b) CNTs:CNFs = 4:1/PDMS/TPU; (c) CNTs:CNFs = 3:2/PDMS/TPU; (d) CNTs:CNFs = 1:1/PDMS/TPU; (e) CNTs:CNFs = 2:3/PDMS/TPU; (f) CNTs:CNFs = 1:4/ PDMS/TPU.

Elongation at break values for the conductive films was assessed using a dynamic fabric resistance tester (FZ01). Experimental data revealed that the baseline elongation at break for the pure TPU film was 200%. Figure 5 illustrates that the elongation at break for sample (a) increased to 214.8%, following the addition of CNTs and PDMS. This increase is attributed to PDMS’s excellent elasticity and flexibility, which facilitates the formation of a robust cross-linking network within the composites, thereby enhancing both ductility and mechanical strength [49,50]. With increased CNF percentages, elongation at break values for samples (b), (c), and (d) improved further to 216.5%, 217.8%, and 217.5%, respectively. However, as the CNF proportion increased further, the elongation at break for samples (e) and (f) decreased to 215.8% and 213.4%, respectively. This decline suggests that excessive CNF can reduce performance due to inadequate dispersion and stress concentration. The addition of an appropriate amount of CNF can effectively enhance the mechanical properties of the films, and the CNT:CNF ratios of 3:2 or 1:1 showed good mechanical properties, making them suitable for strain sensors requiring high ductility.

### 3.5. Hydrophobicity and Corrosion Resistance

**Figure 6:**
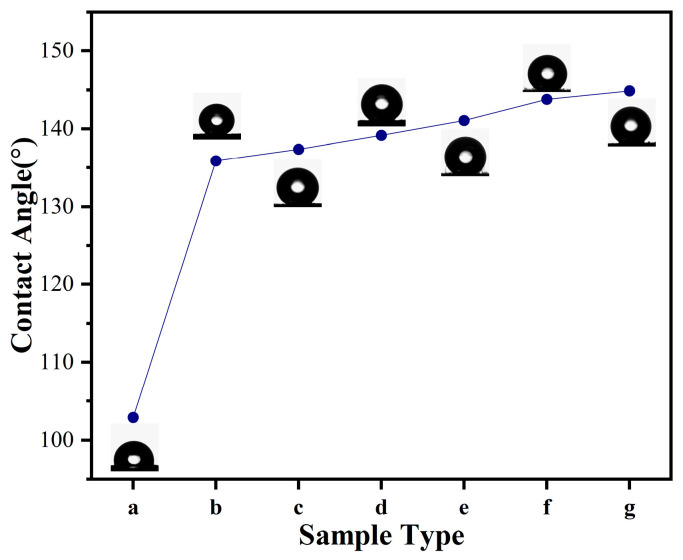
Contact angle of different samples: (a) CNTs/TPU; (b) CNTs/PDMS/TPU; (c) CNTs:CNFs = 4:1/PDMS/TPU; (d) CNTs:CNFs = 3:2/PDMS/TPU; (e) CNTs:CNFs = 1:1/PDMS/TPU; (f) CNTs:CNFs = 2:3/PDMS/TPU; (g) CNTs:CNFs = 1:4/PDMS/TPU.

The contact angle is the angle formed when a liquid droplet comes into contact with a solid surface. It is a visual method to assess the surface properties of a material, especially the hydrophilicity of the surface. As shown in Figure 6, sample (a) is CNT/TPU control without PDMS and CNF incorporation, which has the smallest contact angle of 102.9°. Sample (b), a CNT/TPU/PDMS conductive film, shows a significantly increased contact angle of 135.9° due to the incorporation of PDMS, which has low surface energy. This addition significantly enhances the film’s hydrophobicity, aligning with findings in other studies [51,52]. From sample (c) to sample (g) for CNT/CNF/PDMS/TPU conductive films with different ratios of CNTs and CNFs, the contact angle gradually increases slightly with the increase in the proportion of CNF in the conductive films, and the introduction of CNF changes the microstructure of the films, increases the surface roughness, and thus indirectly enhances the hydrophobicity. Overall, the addition of PDMS and CNFs resulted in significant changes in the surface properties of the materials, especially in enhancing the hydrophobicity.

**Figure 7:**
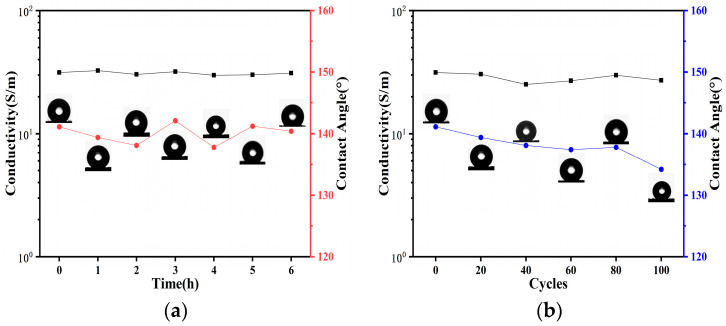
(**a**) Conductivity and contact angle of CNT/CNF/PDMS/TPU films immersed in acidic solution (pH = 1) with time. (**b**) Variation in conductivity and contact angle of CNT/CNF/PDMS/TPU films with the number of cycles at 20% strain.

Given that strain sensors are likely to be exposed to harsh environments such as sweat impregnation in practical applications, this study also explored the performance of conductive films in corrosive conditions. Leveraging the hydrophobic properties of CNT/CNF/PDMS/TPU fiber-based conductive films as demonstrated in Figure 6, systematic tests for corrosion resistance and durability were conducted. The corrosion resistance test was first performed by immersing the films in an acidic solution with pH 1 for 6 h, during which the changes in contact angle and conductivity were monitored as shown in Figure 7a. As immersion time increased, the film’s contact angle consistently remained around 140°, and conductivity was relatively stable. This stability, attributable to the hydrophobic nature of PDMS, effectively prevents corrosive attacks on the film structure, thus demonstrating good corrosion resistance [53].

To assess material durability, the film underwent 100 cyclic stretching tests using a dynamic resistance tester (FZ01) at 20% strain, observing corresponding changes in contact angle and conductivity. As illustrated in Figure 7b, although the contact angle slightly decreased with increasing cycles, it remained around 135°, indicative of sustained hydrophobicity. The films retained their hydrophobic properties under repeated mechanical stress, with minimal changes in conductivity, demonstrating robust durability [54].

### 3.6. Strain Sensitivity Characteristics

The gauge factor (GF) quantifies the sensitivity and responsiveness of nanofiber-based conductive films to strain. This factor primarily assesses how the film’s resistance changes under stretching or compression, serving as a crucial parameter for evaluating its efficacy as a strain sensor [55,56]. The GF of nanofiber-based conductive films under various deformations is calculated from the curve depicting the rate of resistance change relative to tensile strain [57]. The formula for calculating GF is as follows:

(1) $$R_{t}=R-R_{0}\;R_{\varepsilon }=R_{t}/R_{0}\;\varepsilon =\Delta L/L_{0}\;\mathrm{GF}=\Delta R_{\varepsilon }/\Delta_{\varepsilon }$$

*R*_0_ represents the initial resistance of the conductive film, i.e., the resistance when strain is absent; *R* denotes the real-time resistance; *R_t_* signifies the absolute change in resistance; *R_ε_* represents the relative rate of resistance change; *L*_0_ is the initial length of the conductive film; ∆*L* denotes the change in length; and ∆_*ε*_ signifies the change in strain. Using these equations, one can precisely describe and calculate the electrical resistance response of the nanofiber-based conductive film to mechanical deformation, thereby assessing its effectiveness and sensitivity as a strain sensor.

**Figure 8:**
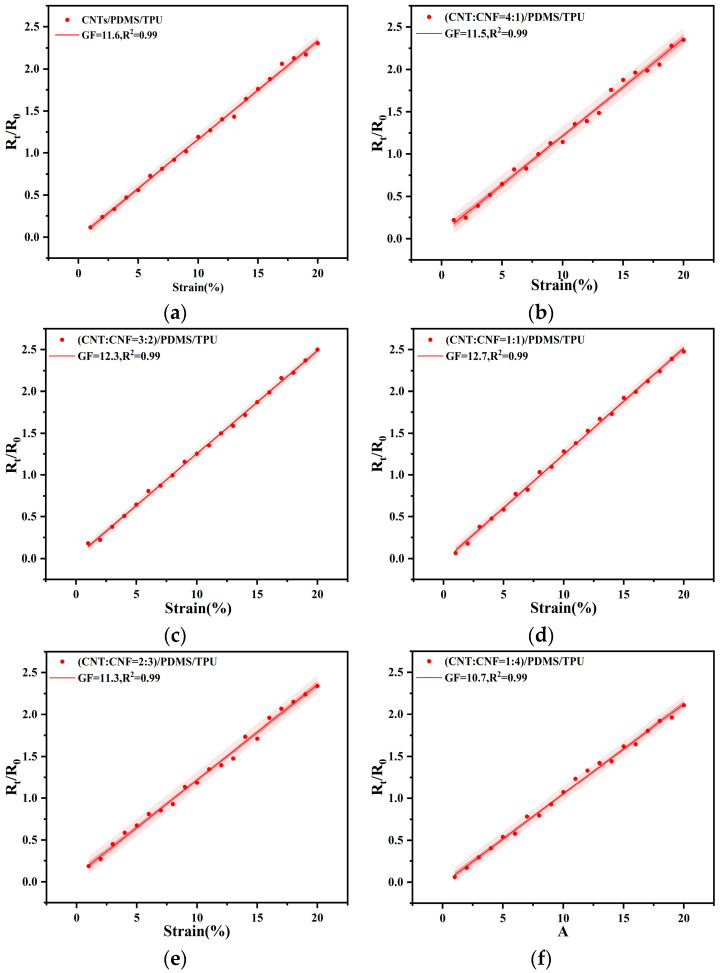
Resistance change versus tensile strain curves for each sample: (**a**) CNTs/PDMS/TPU; (**b**) CNTs:CNFs = 4:1/PDMS/TPU; (**c**) CNTs:CNFs = 3:2/PDMS/TPU; (**d**) CNTs:CNFs = 1:1/PDMS/TPU; (**e**) CNTs:CNFs = 2:3/PDMS/TPU; (**f**) CNTs:CNFs = 1:4/PDMS/TPU.

Fiber-based conductive films were subjected to a 20% tensile strain using a textile dynamic resistance tester (FZ01). Changes in resistance values from 0 to 20% strain were recorded using a digital oscilloscope. As depicted in Figure 8a, the gauge factor (GF) value for CNT/PDMS/TPU films stands at 11.6. Figure 8b–f illustrates the resistivity change versus tensile strain curves for five groups of films with varying blended conductive filler ratios, all of which demonstrate favorable strain sensitivity characteristics. The results indicate that at a CNT to CNF ratio of 1:1, the gauge factor (GF) value peaks at 12.7, signifying that the strain response of the film is optimally sensitive. With further increases in CNF content, notably at CNT:CNF ratios of 2:3 and 1:4, a marked decline in GF value occurs due to the higher CNF content disrupting the conductive network formation, thereby diminishing strain sensitivity. Overall, the conductive films demonstrate optimal strain-sensitive properties at a 1:1 ratio of CNTs to CNFs.

### 3.7. Tensile Cycle Stability

**Figure 9:**
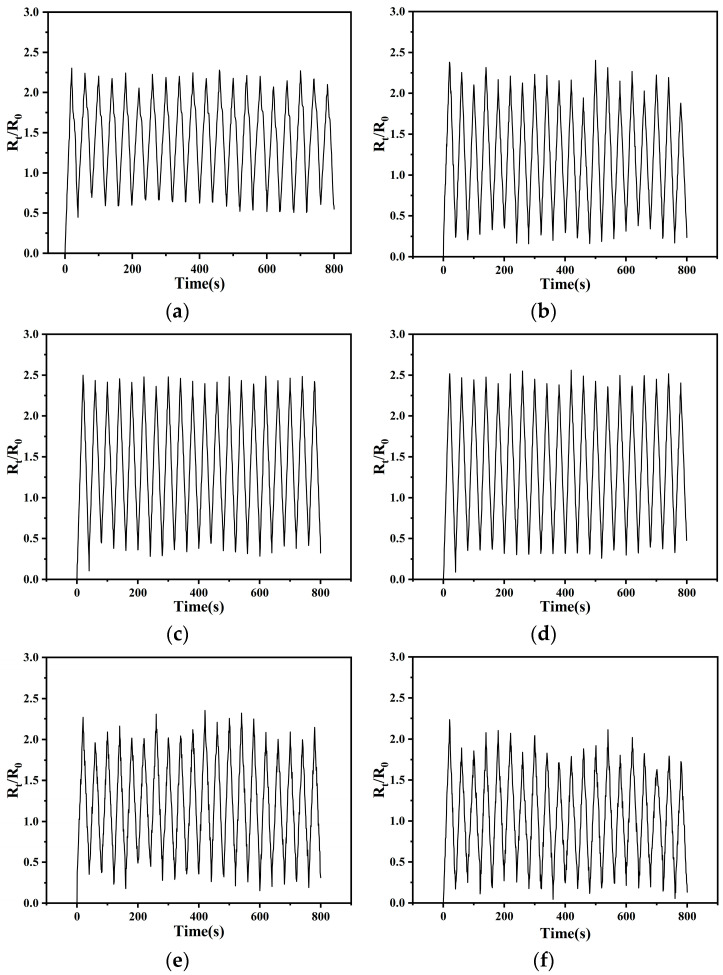
Resistance change in each sample within 20 tensile cycles: (**a**) CNTs/PDMS/TPU; (**b**) CNTs:CNFs = 4:1/PDMS/TPU; (**c**) CNTs:CNFs = 3:2/PDMS/TPU; (**d**) CNTs:CNFs = 1:1/PDMS/TPU; (**e**) CNTs:CNFs = 2:3/PDMS/TPU; (**f**) CNTs:CNFs = 1:4/PDMS/TPU.

In assessing fiber-based conductive films as strain sensors, stability over repeated tensile cycles is a critical aspect, alongside strain sensitivity. This stability indicates the sensor’s capacity to consistently and reliably perform over extended usage or numerous strain actions, essential for ensuring performance in practical applications [52,57,58]. Specifically, it pertains to the consistent rate of resistance change across successive tensile release cycles. To thoroughly examine this characteristic, five films, varying in CNT:CNF ratios, and CNT/PDMS/TPU films, underwent 20 cyclic tensile tests at 20% strain. Figure 9 displays the resistivity change over time for six sets of samples (a–f). Each graph clearly illustrates the fluctuation in the resistive rate of change throughout each stretch–release cycle. Although all samples exhibit periodic resistance changes, differences are noted in peak presence within their resistive rate of change and the degree of fluctuation.

Statistical analysis of the peak resistivity change rate for each sample enabled the calculation of the average and fluctuation rates for the six groups of samples. The average peak resistance change rate quantifies the mean degree of resistance change the film undergoes under specific stress or deformation conditions. A higher value of this index suggests greater material sensitivity to stress or deformation, marking it as a crucial parameter for evaluating the performance of flexible sensors.

The volatility, meanwhile, measures the stability of the peak resistance change rate under repeated stress or deformation conditions. Lower volatility signifies improved repeatability and stability of the material over extended use, crucial for long-term monitoring applications. The volatility *σ*, which is the standard deviation of the peak resistive rate of change, is calculated as follows:

(2) $$\sigma =\sqrt{\frac{\sum_{i-1}^{n}{\left(R_{i}-\bar{R}\right)}^{2}}{n-1}}\times 100\%$$

where *n* is the number of peak occurrences within the cycle, *R_i_* denotes each measured peak, and $\bar{R}$ is the average value of the peak.

**Figure 10:**
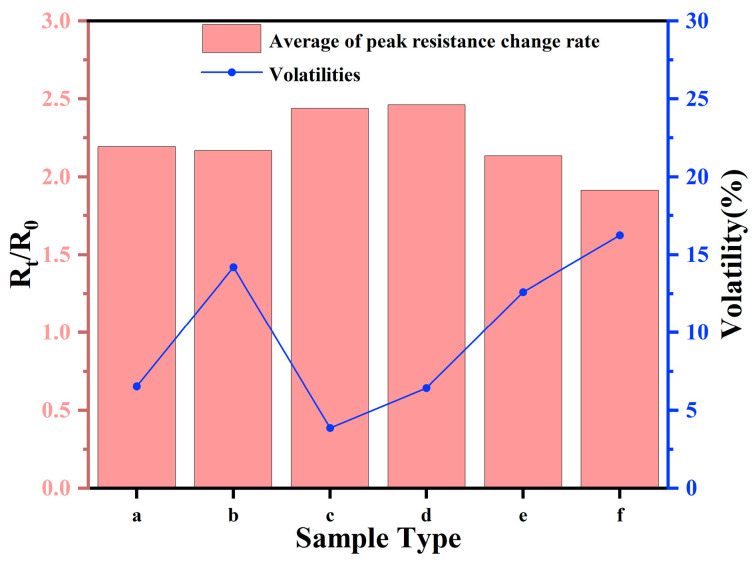
Peak mean and fluctuation of resistance change rate for each sample: (a) CNTs/PDMS/TPU; (b) CNTs:CNFs = 4:1/PDMS/TPU; (c) CNTs:CNFs = 3:2/PDMS/TPU; (d) CNTs:CNFs = 1:1/PDMS/TPU; (e) CNTs:CNFs = 2:3/PDMS/TPU; (f) CNTs:CNFs = 1:4/PDMS/TPU.

The results of these calculations are displayed in Figure 10. As the CNF ratio increases, the peak mean initially rises and subsequently falls, whereas volatility first decreases and then increases. At ratios of 3:2 or 1:1, the synergistic effect of CNFs and CNTs creates a more continuous conductive pathway. This interaction results in superior conductive film performance, both in terms of peak average and volatility in resistance change rate. However, beyond a certain CNF threshold, excessive CNF can tighten or disrupt the structure, diminishing CNT interconnections, and thereby reducing conductive efficiency and strain sensitivity. Upon comprehensive evaluation of the 3:2 and 1:1 ratios, these conductive films were found to possess high sensitivity in peak average resistance change rates and to maintain stability over multiple uses. These characteristics render both ratios ideal for high-performance flexible sensor applications that demand precise and dependable monitoring.

**Figure 11:**
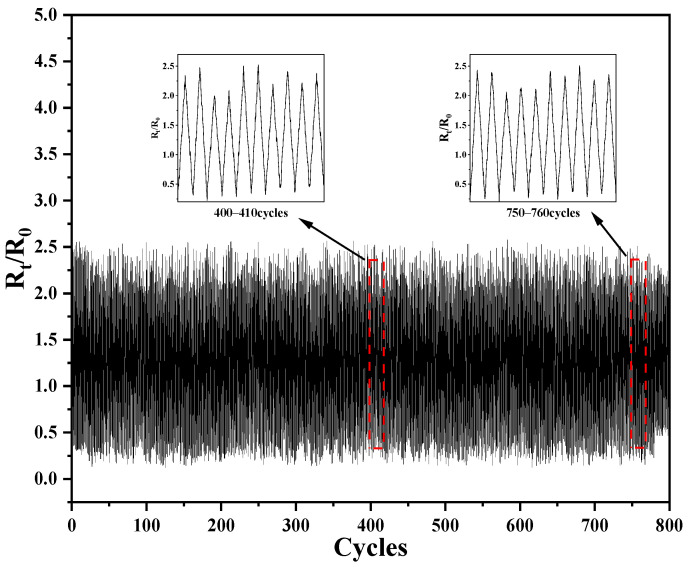
CNTs:CNFs = 1:1/PDMS/TPU conductive film resistance change within 800 cycles at 20% tensile strain.

Figure 11 demonstrates the resistive change rate of CNTs:CNFs = 1:1/PDMS/TPU conductive films over 800 cycles at 20% tensile strain. Analyzing the detailed data from 400 to 410 and 750 to 760 cycles, the conductive films all hold up well, and the results indicate that the resistivity change rate maintains good operational stability over 800 cycles.

In summary, the comprehensive performance analysis of nanofiber-based conductive films with different ratios of blended conductive fillers (CNTs:CNFs = 4:1, 3:2, 1:1, 2:3, and 1:4) reveals that the conductive films prepared with the ratio of CNTs:CNFs = 1:1 have the optimal overall performance in terms of key performance indices such as electrical conductivity, mechanical strength, hydrophobicity, strain sensitivity, and cycling stability. The films with this ratio not only maintained high conductivity and elongation at break but also exhibited the highest strain sensitivity and good cycling stability, indicating an optimal balance between constituting an efficient conductive path and mechanical toughness. Consequently, CNT/CNF/PDMS/TPU nanofiber-based conductive films with a CNT:CNF ratio of 1:1 can be an option as a conductive material for flexible sensors.

### 3.8. CNT/CNF/PDMS/TPU Nanofiber-Based Conductive Films for Human Motion Detection

**Figure 12:**
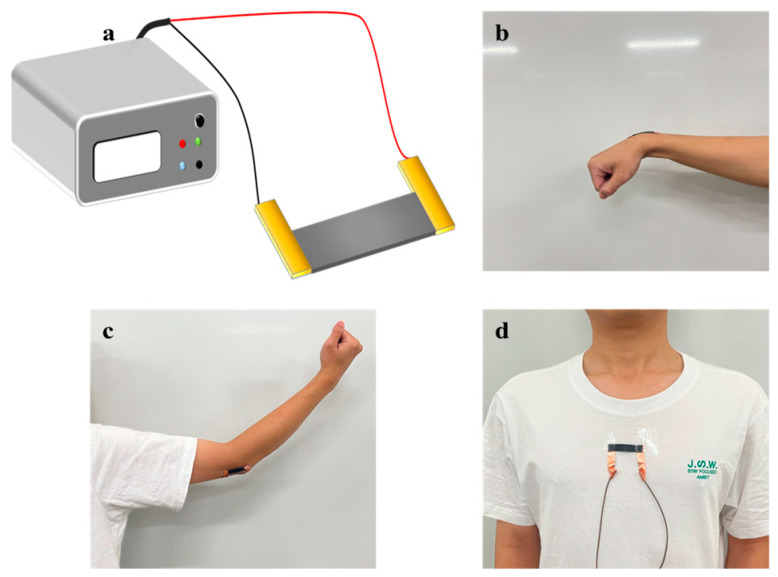
(**a**) Schematic diagram of strain transducer. (**b**) wrist test; (**c**) elbow test; (**d**) chest breathing test.

To verify the potential of CNT/CNF/PDMS/TPU nanofiber-based conductive films for flexible sensors, copper tapes were applied as electrodes at both ends of the films. The copper tape was then connected via wire to a digital oscilloscope to monitor resistance changes, as depicted in Figure 12a. Human movement tests were conducted by attaching the sensors to volunteers’ wrists, elbows, and chests, as illustrated in Figure 12b–d. Designed to simulate everyday movements like bending, stretching, and breathing, these tests assessed the sensor’s stability and responsiveness in real-world applications. A digital oscilloscope facilitated real-time recording of resistance changes across various motion states, evaluating the sensor’s responsiveness to human movement.

**Figure 13:**
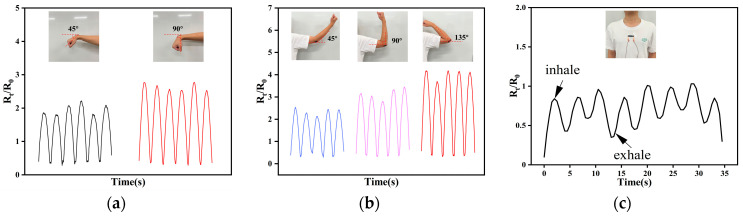
CNT/CNF/PDMS/TPU nanofiber-based conductive films applied to human motion. Resistivity change rate curves: (**a**) Wrist motion. (**b**) Elbow joint motion. (**c**) Chest heave detection during breathing.

In the wrist bending experiments, each wrist was flexed six times to 45° and 90° angles, with results displayed in Figure 13a. As observed with CNT/PDMS/TPU films, the resistive change rate increased with the bending angle, subsequently decreasing upon recovery. The peak resistive change rates fluctuated between 1.8 and 2.2 at a 45° bend and between 2.5 and 2.7 at 90°. Signal stability was maintained throughout the experiment.

Similarly, the elbow underwent five bends at angles of 45°, 90°, and 135°, with outcomes shown in Figure 13b. At a 45° bend, resistance rate fluctuation ranged from 2.1 to 2.5; at 90°, from 2.8 to 3.4; and at 135°, from 3.6 to 4.1, indicating increasing resistance rate changes with greater bending angles. Signal stability was consistent throughout the experiment.

CNT/CNF/PDMS/TPU films also detect thoracic cavity undulations during respiration, as depicted in Figure 13c. Attached to volunteers’ thoracic cavities, these films stretch and peak in resistance change rate upon inhalation and contract with a corresponding decrease upon exhalation. These tests demonstrate the potential of CNT/CNF/PDMS/TPU nanofiber-based conductive films for wearable device applications.

## 4. Conclusions

In summary, this paper introduces a new method based on centrifugal spinning for the preparation of CNT/CNF/PDMS/TPU nanofiber-based conductive films, demonstrating significant potential for strain sensor applications. Initially, TPU nanofiber films were fabricated using the centrifugal spinning method. Subsequently, a proportional mixture of CNTs and CNFs was achieved through the ultrasonic dispersion technique to create the hybrid conductive filler. This filler was then effectively anchored onto the TPU nanofiber-based films using ultrasonic dispersion and impregnation techniques, culminating in surface modification via PDMS impregnation. Analysis of the morphology, composition, and thermal stability of the nanofiber-based conductive films was performed using SEM, FTIR, XRD, and TGA. The nanofiber-based conductive films underwent experimental testing for electrical conductivity, hydrophobicity, corrosion resistance, and tensile cycling. The experimental results demonstrate that under conditions of a CNT to CNF ratio of 1:1, a TPU concentration of 20%, and a PDMS concentration of 2 wt%, the prepared films maintain high electrical conductivity (31.4 S/m) while exhibiting excellent elongation at break (217.5%), robust hydrophobicity and corrosion resistance, high strain sensitivity, and stability over 800 cycles of tensile testing. Furthermore, the CNT/CNF/PDMS/TPU nanofiber-based conductive films demonstrated robust strain response and stability during human motion behavior tests, accurately recording changes in human motion. The CNT/CNF/PDMS/TPU nanofiber-based conductive films, prepared via the centrifugal spinning method, hold significant reference value for the development and application of strain sensors designed to monitor human joint motion and breathing and facilitate large-scale production.

## Data Availability

Due to limitations such as privacy or ethics, data are available upon request. The data presented in this study are available upon request from the corresponding authors. Due to privacy issues involved in the laboratory and the team’s testing process, the data are not made public.
